# Trial protocol: Feasibility of neuromodulation with connectivity-guided intermittent theta-burst stimulation for improving cognition in multiple sclerosis

**DOI:** 10.1515/med-2023-0814

**Published:** 2023-09-28

**Authors:** Caroline Blanchard, Blanca De Dios Perez, Tierney Tindall, Katie Clarkson, Ghadah Felmban, Grit Scheffler-Ansari, Roger Periam, Sudheer Lankappa, Cris S. Constantinescu, Roshan das Nair, Richard Morriss, Nikos Evangelou, Dorothee P. Auer, Rob A. Dineen

**Affiliations:** Radiological Sciences, Academic Unit of Mental Health and Clinical Neuroscience, University of Nottingham, Nottingham, United Kingdom; NIHR Nottingham Biomedical Research Centre, Nottingham, United Kingdom; Centre for Rehabilitation and Ageing Research, School of Medicine, University of Nottingham, Nottingham, United Kingdom; School of Psychology and Vision Sciences, University of Leicester, Leicester, UK; School of Applied Medical Sciences, King Saud Bin Abdul-Aziz University for Health Sciences, Riyadh 14611, Saudi Arabia; Patient and Public Involvement (PPI) Representatives., c/o Academic Unit of Mental Health and Clinical Neuroscience, University of Nottingham, Nottingham, United Kingdom; Nottinghamshire Healthcare NHS Foundation Trust, Nottingham, United Kingdom; Clinical Neurology, Academic Unit of Mental Health and Clinical Neuroscience, University of Nottingham, Nottingham, United Kingdom; Cooper University Hospital, Cooper Neurological Institute, Cooper Medical School at Rowan University, Camden, NJ, 08103, USA; Institute of Mental Health, University of Nottingham, Nottingham, United Kingdom; Department of Health Research, SINTEF, Trondheim, Norway

**Keywords:** neuromodulation, transcranial magnetic stimulation, intermittent theta-burst stimulation, cognition, multiple sclerosis

## Abstract

Cognitive impairment in multiple sclerosis (MS) can adversely impact participation in employment, activities of daily living, and wider society. It affects 40–70% of people living with MS (pwMS). There are few effective treatments for cognitive impairment in people with MS. Neuromodulation with intermittent theta-burst stimulation (iTBS) has potential for treating cognitive impairment in pwMS. This single-centre mixed-methods feasibility randomised controlled trial (NCT04931953) will assess feasibility, acceptability, and tolerability of procedures used for applying iTBS for improving cognitive performance in pwMS. Participants will be randomised into three intervention groups with varying lengths of iTBS treatment (from 1 to 4 weeks) and a sham-control group. Quantitative data will be collected at three time points (baseline, end of intervention, and 8-week follow-up). End of the intervention semi-structured interviews will explore the views and experiences of the participants receiving the intervention, analysed using framework analysis. Quantitative and qualitative data will be synthesised to explore the impact of the iTBS intervention. Ethical approval has been received from the Health Research Authority (21/LO/0506) and recruitment started in June 2022. The results will inform the design of an RCT of the efficacy of iTBS as a therapeutic intervention for cognitive impairment in pwMS.

## 1 Introduction

Multiple sclerosis (MS) is a chronic inflammatory demyelinating and neurodegenerative condition of the central nervous system [1] that affects around 140,000 people in the United Kingdom [2] and is a common cause of physical disability among working-age adults [3]. Cognitive impairment affects up to 70% of people with MS which can adversely impact participation in employment, activities of daily living, social interaction, and the individual’s potential to engage with rehabilitation [4]. There are currently few effective treatments for cognitive impairment in people with MS [5,6]. A recent expert position paper has called for the development of treatments that are based on biologically plausible mechanisms of action and that these are trialled in high-quality trials with outcome measures that are meaningful in the context of the patient’s life [7].

Interventions that can directly modulate the function of targeted brain regions have been shown to reduce the burden of symptoms in depression [8] and have potential therapeutic roles in Parkinson’s disease [9] and Alzheimer’s disease [10]. One such neuromodulation approach is repetitive transcranial magnetic stimulation (rTMS), a non-invasive procedure that applies magnetic pulses on the surface of the scalp to reach underlying brain tissue [11]. rTMS is reported as a safe and effective treatment, does not require hospital admission or anaesthesia, and is approved by the National Institute for Health and Care Research for the treatment of depression [12] and migraine [13].

Repetitive TMS can increase the excitability in the targeted cortical (and connected) brain regions. However, there has been evidence showing that high-frequency rTMS poses some side effects including seizure, headache, and neck pain. Intermittent theta-burst stimulation (iTBS) is a type of rTMS that uses short bursts of three high-frequency pulses administered with a short inter-burst interval that mimics the activity (firing rate) of specific types of neurons and reduces the administration time. iTBS potentiates plasticity of brain circuits in healthy individuals that lasts for a longer duration than the changes induced by standard rTMS protocols [14].

A recent systematic review found reported improvements in working memory and Stroop task performance in predominantly young (mean age 26 years) healthy non-cognitively impaired volunteers receiving iTBS targeting the prefrontal cortex [15]. Furthermore, comparable effects on brain physiology can be obtained by a lower dose of iTBS that lasts for a shorter duration than standard TMS as the required number of pulses gets delivered in a shorter time [16]. iTBS is shown to be as safe as the standard TMS protocol and appears to have a low incidence of adverse effects [16].

The neural circuit including the dorsolateral prefrontal cortex (DLPFC) is reported to have a role in regulating executive functions [17,18,19]. Studies have found evidence that people with MS reporting cognitive dysfunction have regional damage in the dorsolateral prefrontal circuit (e.g., striatum, globus pallidus, thalamus), including atrophied DLPFC, caudate, or thalamus [20,21,22]. In an analysis of 52 people with MS and 36 non-MS controls, we found that effective connectivity of the DLPFC to the caudate nucleus was decreased in people with MS who had impaired performance in the Paced Auditory Serial Addition Task 3-second stimulus (PASAT-3) compared to people with MS without PASAT-3 performance impairment and non-MS participants [23]. The findings highlight the DLPFC as a possible target for interventions aiming to maintain and/or improve cognitive functioning for people with MS through modulation of effective connectivity [23].

## 2 Methods

### 2.1 Study aim

This study will test the feasibility and acceptability of iTBS as an intervention for improving cognitive performance in people with MS. Group allocation will allow comparison of feasibility and acceptability of iTBS administration schedules of different total durations (1, 2, and 4 weeks) and assessment of the believability of the sham administration, which will inform the design of a phase III trial which will investigate the clinical- and cost-effectiveness of the treatment.

### 2.2 Study design

A single-centre, mixed-methods feasibility randomised controlled trial will be conducted to compare four groups (target: 10 participants each) of iTBS administration. Participants will be randomised into one of four groups:

- Group 1: iTBS intervention – daily sessions for 4 days over 1 week (total 4 iTBS sessions).
- Group 2: iTBS intervention – daily sessions for 4 days per week over 2 weeks (total 8 iTBS sessions).
- Group 3: iTBS intervention – daily sessions for 4 days per week over 4 weeks (total 16iTBS sessions)
- Control Group 4: sham iTBS intervention – daily sessions for 4 days per week over 2 weeks (total 8 sham iTBS sessions).

Participants will complete outcome measures at three time points during the study: baseline, end of intervention (EOI) (i.e., 5 weeks after the baseline), and 8-week follow-up (i.e., 13 weeks after baseline). Participants will undergo MRI scans at baseline and EOI. We will conduct post-intervention interviews to explore the participants’ experiences of the trial, including the tolerability of the protocol, acceptability of the visit schedule, and any perceived differences in cognition.

#### 2.2.1 Primary objective

The primary objective is to assess the feasibility of the trial procedures, in terms of their acceptability and tolerability for people with MS.

To address this primary objective, we will:

– Assess the feasibility of recruitment, including the proportion of eligible and consenting participants.
– Assess the acceptability of overall trial participation for the 1-, 2-, and 4-week groups.
– Assess the tolerability of the iTBS intervention (or sham), including any side effects.
– Establish the believability of sham iTBS administration.

#### 2.2.2 Secondary objectives

Secondary objectives are to:

– Measure changes in cognitive assessment scores by group between baseline and EOI assessments (standardised interval of 5 weeks apart), to allow comparison of effect sizes between groups.
– Measure changes in cognitive assessment scores by group between EOI and 8-week follow-up assessments, to allow comparison of effect sizes between groups.
– Measure changes in participant-reported effects on cognition by group between baseline, EOI, and 8-week follow-up assessments, to allow the comparison of effect sizes between groups.
– Explore (at the follow-up interview) participant views on meaningful change in cognition in relation to the demands of trial participation.
– Measure changes in scores for anxiety, depression, and fatigue before and after intervention, to allow comparison of effect sizes between groups.
– Measure changes in the effective connectivity between left DLPFC and left caudate nucleus, to allow correlation with changes in cognition.

### 2.3 Eligibility criteria

#### 2.3.1 Inclusion criteria

– Clinical diagnosis of MS (any type of MS) according to the 2017 McDonald criteria [24] at least 12 months prior to baseline assessment.
– Score of 55 or lower on the oral Symbol Digit Modality Test (SDMT). The SDMT threshold of 55 or lower has been chosen because it is 1 standard deviation (1sd) below the mean for young adults with high educational level, and this range includes the respective cut-offs for 1sd below mean values for other age and educational groups. This lenient cut-off value is not intended to select individuals with cognitive impairment *per se*, but to remove high-performing individuals [25]. This allows recruitment of an inclusive group of people who *may* have cognitive impairment, with a range of lower cognitive performance levels, for the purpose of this feasibility study.
– Aged between 18 and 69 years.
– Ability to give informed consent.
– Able to commit to regular attendance in the clinic, for up to 4 times a week for 4 weeks and follow-up assessment 8 weeks after the end of trial procedures.

#### 2.3.2 Exclusion criteria

– Depressive symptoms present with a score of ≥15 (the threshold for moderate depression) on the Patient Health Questionnaire-9 (PHQ-9) [26].
– Medical history of, or self-reported, seizures.
– Co-morbid neurological conditions (in addition to MS), e.g., brain neoplasm, cerebrovascular events, epilepsy, prior brain injury, or brain surgery.
– Contraindications to MRI scanning (identified by standard MRI safety screening questionnaire).
– Contraindications to TMS, including hairstyles or piercings (which cannot be altered) that would impair magnetic pulse delivery.
– Frequent panic attacks which are likely to prevent regular attendance or participation in MRI/TMS procedures.
– Prior TMS intervention.
– Pregnancy.
– MS relapse within the preceding 6 weeks.
– Significant mobility problems if they are likely to preclude regular attendance in the clinic, for up to 4 times a week for 4 weeks.
– Involved with any other clinical trials involving medical procedures, interventions, or treatment.
– Not fluent English speaker hampering the completion of questionnaires and cognitive assessments.

### 2.4 Recruitment, consent, randomisation, and blinding

**Figure 1:**
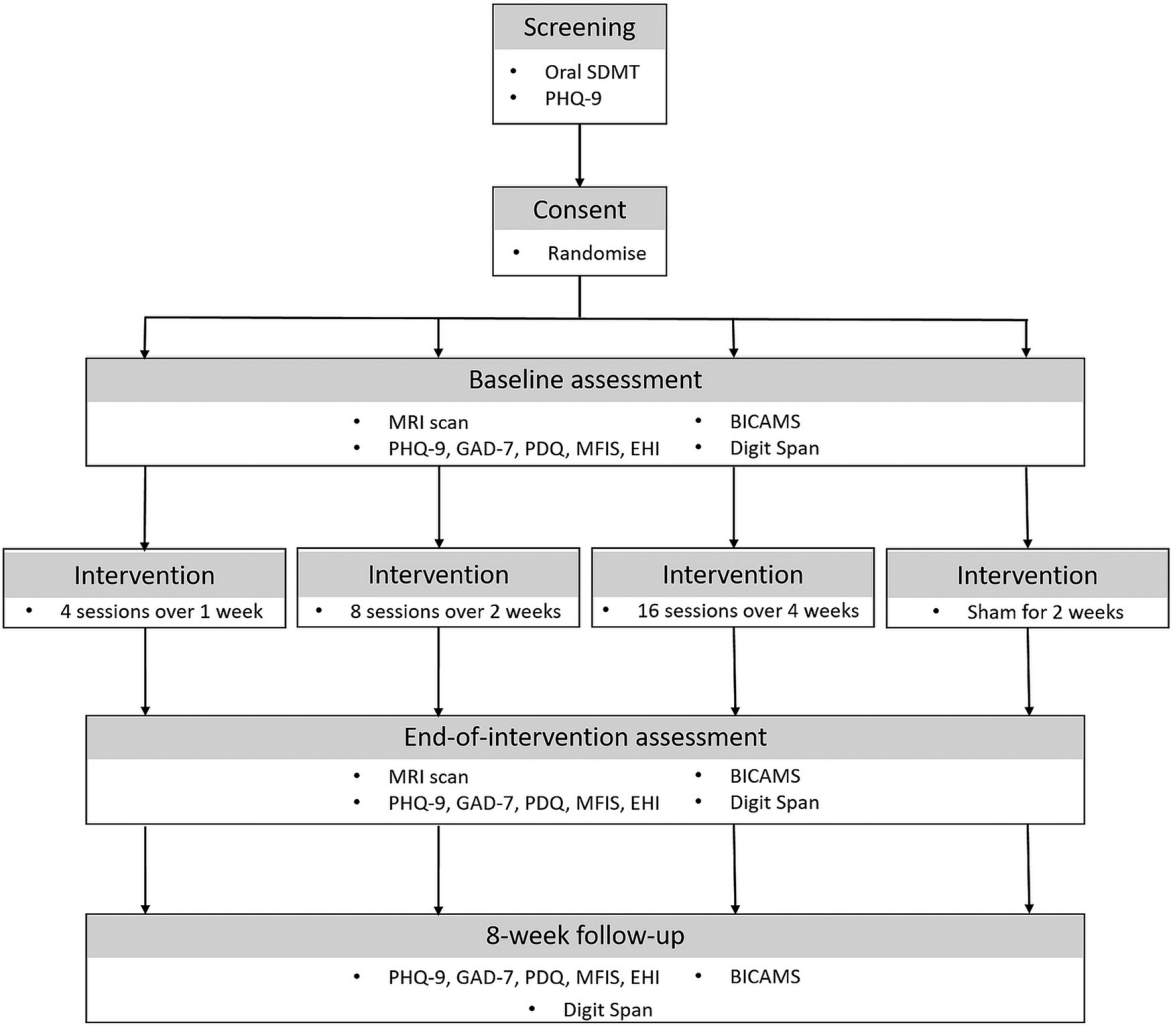
Trial flowchart.

Participant flow through the trial is shown in Figure 1. Participants will be recruited from the MS Clinic at the Queens Medical Centre (Nottingham, UK) and will initially be approached by a member of the patient’s usual care team. Potential participants may also contact the research team after seeing publicly available information about the study (e.g., social media, newsletters, posters). Potential participants who express an interest in the study will be sent a participant information sheet presenting information about the study and will have the opportunity to contact a member of the research team via telephone or email to ask further questions. Participants will be recruited on a “first come, first served” basis.

Consenting procedures will be conducted by the research psychologist. Participants will be randomised after completing the consent form, and the research psychologist will be blinded to group allocation. Participants will be randomised to one of four groups in equal proportions (1:1:1:1 ratio) automatically using blocked stratified randomisation, stratified on: age group (≤40), gender (man or woman), and MS type (relapsing-remitting MS or secondary progressive or primary progressive MS). The randomisation schema was created on 9th November 2021 10:51 using *ralloc.ado* version 3.5.2 [27] in Stata version 17. The neuromodulation research fellow is notified of the group allocation via an automatic email. The research psychologist and principal investigator will remain blinded to group allocation. The two TMS operators (including the neuromodulation research fellow) administering the iTBS will not be blind to group allocation.

**Figure 2:**
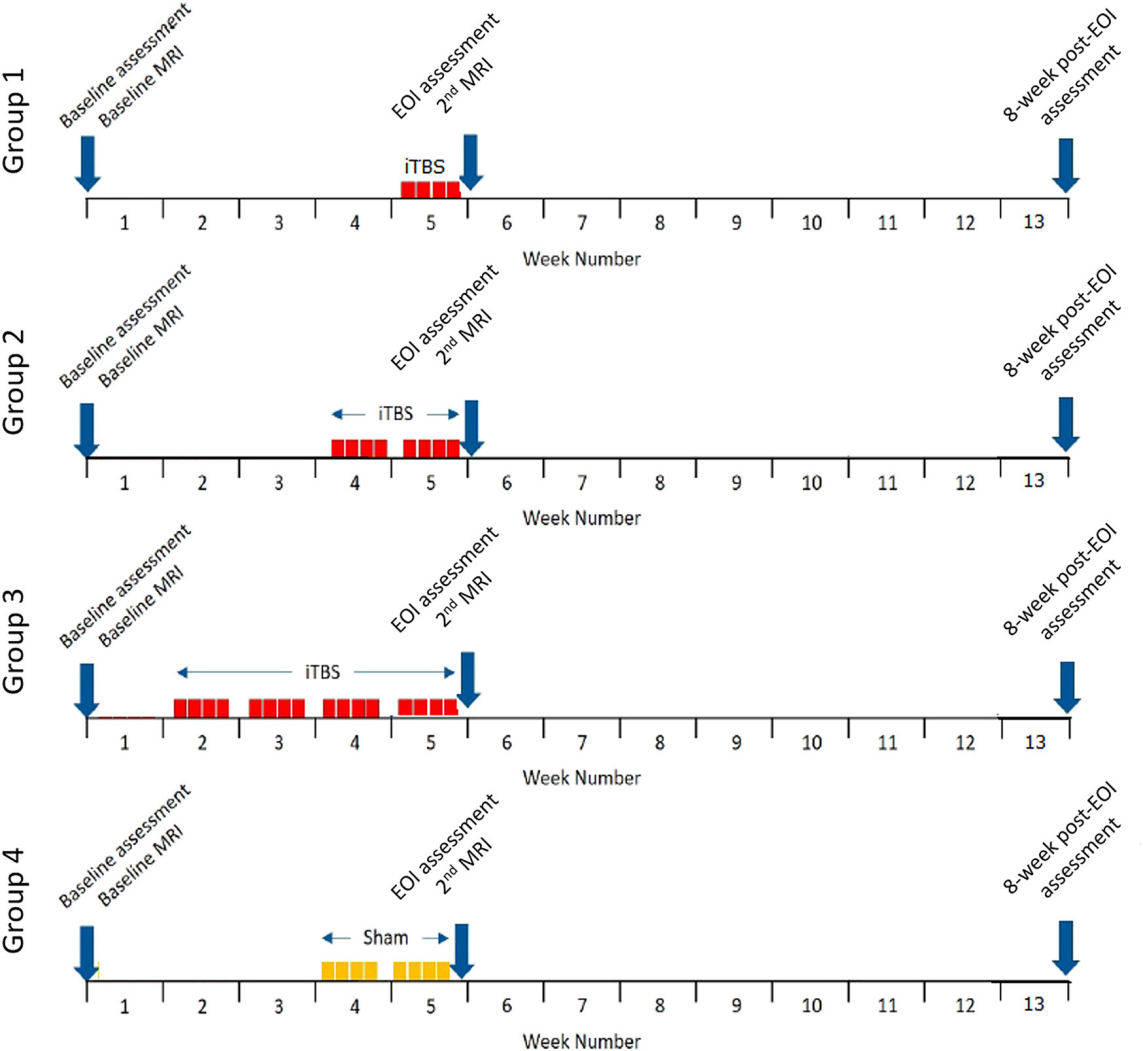
Intervention and assessment schedule per group allocation.

Participants will be blinded as to whether their iTBS intervention is real (groups 1, 2, and 3) or sham (group 4) until the end of their study participation. Although only the 2-week intervention duration has a sham comparator (group 2 = real iTBS, group 4 = sham iTBS), participants will not be made aware that the 1- and 4-week intervention durations do not have a sham comparator. Participants will be instructed not to tell the research psychologist the duration of their intervention, as this would reveal to the research psychologist that the patient received the real iTBS intervention if in the 1- or 4-week intervention groups. To standardise the assessment interval and maintain the blinded status of the research psychologist, the interval between baseline and EOI assessments will be fixed at 5 weeks, requiring commencement of the iTBS intervention to be staggered per group as per Figure 2.

### 2.5 Intervention

Depending on the group, participants will receive iTBS (or sham) intervention 4 times per week for 1, 2, or 4 weeks. Each intervention session will last around 30 min, including 20 min of iTBS. The iTBS (or sham) sessions will be delivered by two TMS operators that completed 15 h of training to deliver TMS. The sessions will be conducted in a private and quiet room located at the Clinical Research Facility of Nottingham University Hospitals. The first intervention session will be scheduled for weeks 2, 4, or 5 depending on the group. We designed this study to have, for all participants regardless of group allocation, a 5-week standardised gap between baseline assessment on week 1, administrated before any iTBS session and the EOI assessments on week 5, administrated immediately after the last iTBS session.

#### 2.5.1 iTBS intervention

For participants allocated to one of the real iTBS groups, stimulation will be administered to the DLPFC target coordinates (see below for target identification) under StimGuide neuronavigation using a 70 mm Double Air Film Coil (Magstim, Whitland, Dyfed, UK), connected to a Magstim Horizon Performance stimulator. The administration comprises bursts of 3 pulses at 50 Hz with a power of 80% of the resting motor threshold (RMT), at a burst frequency of 5 Hz (i.e., every 200 ms) for 2 s, repeated every 10 s for a total of 190 s (600 pulses) [28]. Blocks will be repeated a total of 3 times, with 5-min rest intervals between blocks, a total of 1800 pulse per treatment session. During left DLPFC stimulation, the iTBS coil is held by support tangentially to the skull, with the axis of the coil angled approximately 45° from the midsagittal plane. The total duration of each administration is approximately 20 min.

#### 2.5.2 Sham intervention

The sham iTBS administration will be performed under the same conditions and with an identical protocol including 50% RMT and equipment to the full administration, except that the 8-shaped iTBS coil is held orthogonally to the scalp surface, its side touching the scalp to provide a similar sensation of direct contact to the real intervention, with the axis of the coil parallel to the midsagittal plane. The centre of the coil is kept roughly 10 cm from the skull and does not deliver any stimulation in the direction of the cortical surface. The sound made by the iTBS coil is the same as the real intervention stimulation. The TMS operators display a sham coil position on the screen of the neuronavigation system, similar to that shown for the real intervention.

### 2.6 Study procedures and data collection

Study data will be collected and managed using REDCap electronic data capture tools [29,30] hosted at the University of Nottingham.

#### 2.6.1 Initial participant screening

Screening procedures will be conducted via telephone or videoconference by the research psychologist. The oral version of the SDMT and the PHQ-9 will be administered. Participants will be asked to complete a questionnaire regarding their demographics, MS diagnosis, and current treatments and to identify possible contraindications to MRI and TMS procedures.

Patients fulfilling the eligibility criteria will be invited to participate in the trial. Those who do not meet eligibility criteria will be excluded and thanked for their time. Reasons for ineligibility will be recorded.

#### 2.6.2 Demographic and clinical characteristics

Following enrolment, missing information regarding the participants’ clinical characteristics (e.g., type of MS, years with MS, years with symptoms suggestive of MS, medications) will be requested from the neurologist or usual MS care team, along with the participants’ most recent Expanded Disability Status Score [31]. The use of disease-modifying treatment by recruited participants will be recorded and reported.

#### 2.6.3 Baseline assessments and MRI scan

All participants regardless of group allocation will complete the following procedures at baseline assessment in week 1.

Participants will undergo in-person baseline cognitive and mood assessment that includes:

1. The Brief International Cognitive Assessment for MS (BICAMS): The BICAMS was developed by an international panel to support a cognitive assessment that is brief and universal, to include tests of mental processing speed and memory. It was developed to be optimised for small centres where staff may not have formal neuropsychological training [32]. Tests included are:

- The SDMT, assessing attention, working memory, and visual/spatial information processing speed [33].
- The California Verbal Learning Test-II, assessing verbal memory recall [34].
- The Brief Visuospatial Memory Test Revised, assessing visual memory [35].

1. Digit Span (from WAIS-IV): this test comprises two conditions, namely Digit Span Forwards (DSF) and Backwards (DSB). DSF assesses attention, encoding, and auditory processing capacity while DSB assesses working memory and executive function [36].
2. Patient Health Questionnaire (PHQ-9) is a module of the PHQ for recording severity of depressive symptoms [26] and has been validated in primary care populations [37,38].
3. Generalised Anxiety Disorder (GAD-7) is a brief scale to screen for generalised anxiety disorder [39].
4. The Perceived Deficits Questionnaire (PDQ) assesses subjective cognitive dysfunction in people with MS [40]. The PDQ includes four subscales (attention/concentration, retrospective memory, prospective memory, and planning/organization).
5. The Modified Fatigue Impact Scale (MFIS) is a self-report questionnaire exploring the effects of fatigue across physical, cognitive, and psychosocial modalities. It is a modified form of the Fatigue Impact Scale [41] and was developed following interviews with people with MS who discussed how fatigue had impacted their daily lives.
6. The Edinburgh Handedness Inventory (EHI) assesses whether participants favour their left or right hand to inform the MRI analysis.

Participants will have a 3T MRI scan to allow target localisation, quantification of MS lesion burden, and characterisation of brain networks and connectivity. The MRI scan will include:

- High-resolution T1-weighted volume, for image co-registration and segmentation;
- Resting-state functional MRI, for functional connectivity analysis;
- Fluid-attenuated inversion recovery, for MS T2 lesion quantification;
- Diffusion tensor imaging, for structural connectivity analysis;

The T1-weighted and resting state functional MRI will be used to estimate the iTBS intervention targets.

#### 2.6.4 Intervention visits

Two working days before the participants’ first visit, staff delivering the iTBS (or sham) intervention will ask participants if anything has changed since they were assessed which may impact their cognitive abilities, such as infection or MS relapse. If they have MS relapse or active/recent infection, they will be withdrawn from the study.

The first iTBS (active or sham) session will begin with the estimation of the RMT as detected using electromyography (EMG) to record a motor response in the contralateral abductor pollicis brevis. This is achieved by administration of single pulses of TMS to the scalp over the approximate location of the primary hand motor area of the left hemisphere, identified using the StimGuide neuronavigation system to locate the C3 position on the scalp. Slight adjustments to the position of the coil and the angle relative to the scalp surface are made by the operator to optimise stimulation of the primary hand motor area. The TMS operator delivers single TMS pulses with varying intensities guided by the adaptive Parameter Estimation by Sequential Testing [42] to estimate the participant’s RMT, defined as the minimum TMS intensity sufficient to produce a >50 μV motor‐evoked potential recoded by EMG in at least 50% of trials [43].

The localisation of the left DLPFC target will be identified using a similar target-identification methodology to previous work [23,44], using effective connectivity of the left caudate to identify the maximally connected locus in the left DLFPC.

The participant then receives the intervention according to group allocation as described above in the ‘Intervention’ section.

Following each administration, the participant is asked to report any sensations they may have experienced.

In case of missed appointments, the research team will work with the participant to reschedule the appointment, providing that participants do not have more than four consecutive days without intervention (including weekends) and that the total afforded extra time does not exceed 50% of the initial intervention schedule.

Thus, participants allocated to 1 week of intervention (group 1) will be allowed 3 extra days within which to complete the intervention schedule. Participants allocated to 2 weeks of intervention (groups 2 and 4) will have one extra week to complete it and participants allocated to 4 weeks of intervention (group 3) will be afforded up to 2 weeks during which to complete it. If the intervention cannot be administrated during the allowed time, this will be recorded as a protocol deviation.

#### 2.6.5 EOI assessment

Participants will undergo repeat cognitive assessments and all questionnaires (except for the EHI) as per the baseline assessment. Participants will have a 3 T MRI scan with an identical protocol to the baseline MRI. Participants will be asked to complete an in-house questionnaire exploring the experience and tolerability of the iTBS protocol and MRI scan.

#### 2.6.6 8-Week follow-up

At 8-week follow-up, participants will complete again the cognitive assessments and questionnaires (except for the EHI), followed by a semi-structured interview exploring the acceptability and tolerability of the intervention and any self-reported changes in cognitive ability experienced in daily life. The interview will also explore participants’ views on what a sufficiently meaningful and durable change in cognitive performance is to balance and justify the demands of the intervention (i.e., daily hospital visits, possible discomfort, etc.) Participants will be asked for suggestions for improving the trial experience.

#### 2.6.7 Early withdrawal interviews

As this is a feasibility trial, participants withdrawing from the study before completing the protocol will be invited for an exit interview to explore the reasons for withdrawal.

### 2.7 Endpoints measured

As this is a feasibility study several endpoints will be collected to allow primary and secondary aims to be assessed, shown in Table 1.

**Table 1:** Feasibility trial endpoints

Endpoints to address the primary objective
Proportion of screened potential participants eligible for participation
Proportion of eligible potential participants consenting for participation
Number of participants completing the visit schedule as per protocol
Questionnaire ratings of tolerability of the iTBS or sham administration
Questionnaire ratings of overall acceptability of trial participation
Number and type of adverse events
Participant opinion at EOT of whether they received real or sham iTBS
**Endpoints to address the secondary objectives**
Scores and test sub-scores in the Brief International Cognitive Assessment for Multiple Sclerosis (BICAMS) test battery
Self-reported cognitive dysfunction: Perceived Deficits Questionnaire (PDQ)
Self-rated mood: Patient Health Questionnaire – Depression (PHQ-9) and Generalised Anxiety Disorder 7 (GAD-7)
Self-reported fatigue: Modified Fatigue Impact Scale (MFIS)
Subjective perceived differences in cognitive abilities in daily life (explored at interview)
Reflections on meaningful changes to cognition in relation to the demands of the trial participation (explored at interview)
Change in effective connectivity between left DLPFC and left caudate nucleus

### 2.8 Sample size

We aim to randomise up to 40 participants (up to 10 participants per group), which should provide sufficient information to inform our feasibility objectives and to inform the design of a potential phase III RCT. Whitehead et al. [45] recommend that pilot trial sample sizes should be between 10 and 20 participants per intervention group, to offer 80% power and two-tailed 5% significance level for standardised effect sizes of small (0.2) or medium (0.5). Therefore, with our acknowledgement that the study procedures may be taxing for people with MS, we have selected the smaller target of 10 participants per group to minimize inconveniences for a wider sample, with respect to our primary objective.

### 2.9 Data analysis plan

Data will be reported according to the CONSORT 2010 statement, extension for pilot and feasibility trials [46]. The research psychologist will perform the scoring cognitive tests and questionnaires scores and collation/tabulation of other endpoints prior to unblinded group analysis. Quantitative data will be analysed using SPSS version 24.0 [47]. We will provide descriptive statistics regarding the characteristics of the participants. Rates of consenting participants, recruitment, intervention completion, acceptability, tolerability, and adverse events will be reported by group on an intention-to-treat analysis (excluding participants who withdrew from the trial before the first iTBS administration).

Quantitative analysis will be used to evaluate the change in cognition, mood, and fatigue using the cognitive assessment and questionnaires. Where appropriate, parametric or non-parametric statistics will be used to estimate effect sizes and perform between-group comparisons to inform power and sample size calculations for a potential future phase III trial.

Qualitative data (post-intervention interviews) will be analysed using framework analysis [48–51]. These will be audio-recorded and transcribed verbatim by the research psychologist. The interview transcripts will be mapped onto a predefined framework. An analytical framework will be developed according to the interview schedule which will map onto the aims of the qualitative study. The researcher will read the transcripts to get familiar with the content of the interview. Subsequent analysis will be conducted on NVivo software [52]. This process will follow Gale et al.’s guide to framework analysis [48]. Following the initial coding of the first transcript, two researchers will meet to discuss the codes and to check whether the coding is consistent and comprehensive. Disagreements will be resolved through discussion together or with a third member of the study team to prevent bias. Once all the coding is completed, the researcher will enter these data onto the framework matrix and determine the fit of the data to this matrix. Any new data that does not fit the matrix will be considered again to see whether a new thematic structure is needed to include such data.

#### 2.9.1 Data synthesis

Quantitative and qualitative data will be synthesised following the convergence coding matrix strategy [53,54]. The research team will complete the quantitative and qualitative analysis separately, and then use a convergence coding matrix to synthesise the findings.

The research team will identify the key factors that provide insight regarding the impact and acceptability of the intervention. The research team will then look for agreement or disagreement between the findings from the different research methods.

The convergence coding matrix follows four possible codes [53,54]:

- Convergence: Agreement between the data from both methodologies.
- Complementary: The findings from one methodology complement the rest of findings.
- Disagreement: The findings from each methodology contradict each other.
- Silence: Only one methodology offers information of a key finding.

### 2.10 Patient and public involvement

Patient and Public Involvement (PPI) representatives have been involved from the inception of the project. This project was presented and discussed at the Nottingham MS PPI group meeting in March 2019. Attendees were asked to rate the relevance of the study and methods used; the attendees believed that the study was highly relevant, and the planning was excellent.

The research team also includes two PPI representatives who contributed to the development of the grant proposal and are involved in the review of participant-facing material, research team meetings to discuss progress, and commented on manuscripts for appropriateness of language.

### 2.11 Dissemination

The findings from this study will be disseminated to relevant stakeholders (e.g., people with MS and their families, healthcare professionals). A main manuscript reporting the feasibility of delivering the proposed intervention and outcomes will be submitted for publication. The findings will also be disseminated at national and international conferences to reach a wider academic audience. Finally, we will share the findings with organisations that work with people living with MS (e.g., MS Society) by writing a lay summary report or presenting at conferences organised by and for people living with MS (e.g., MS Life conference)

### 2.12 Trial status

Recruitment commenced on 22 June 2022. At present, 32 potential participants have been screened, 24 have been recruited, 14 have completed the intervention (or sham), and 13 have completed the 8-week follow-up assessment.


**Ethical approval:** The study received a favourable ethical opinion from the Health Research Authority (ref: 21/LO/0506) on 20 August 2021. The study is registered with ClinicalTrials.gov (NCT04931953).

## 3 Conclusion

This manuscript reports the study protocol for mixed-methods feasibility randomised controlled trial exploring the feasibility and acceptability of iTBS improving for cognitive performance in people with MS. Data regarding the feasibility and acceptability of the intervention will be collected using mixed methods research to allow for an in-depth understanding of the impact of the intervention and identify barriers for future implementation in a larger phase III RCT.
